# The Combined ICP-MS, ESEM-EDX, and HAADF-STEM-EDX Approach for the Assessment of Metal Sub-Micro- and Nanoparticles in Wheat Grain

**DOI:** 10.3390/molecules29133148

**Published:** 2024-07-02

**Authors:** Maurizio Piergiovanni, Monica Mattarozzi, Eveline Verleysen, Lisa Siciliani, Michele Suman, Federica Bianchi, Jan Mast, Maria Careri

**Affiliations:** 1 Department of Chemistry, Life Sciences and Environmental Sustainability, University of Parma, Parco Area delle Scienze 17/A, 43124 Parma, Italy; maurizio.piergiovanni@unipr.it (M.P.); federica.bianchi@unipr.it (F.B.); 2 Interdepartmental Center on Safety, Technologies and Agri-Food Innovation (SITEIA.PARMA), University of Parma, Parco Area delle Scienze 181/A, 43124 Parma, Italy; 3 Service Trace Elements and Nanomaterials, Sciensano, Groeselenbergstraat 99, 1180 Uccle, Belgium; eveline.verleysen@sciensano.be (E.V.); lisa.siciliani@sciensano.be (L.S.); jan.mast@sciensano.be (J.M.); 4 Advanced Laboratory Research, Barilla G. e R. Fratelli S.p.A., Via Mantova, 166, 43122 Parma, Italy; michele.suman@barilla.com; 5 Department for Sustainable Food Process, Catholic University Sacred Heart, via Emilia Parmense 84, 29122 Piacenza, Italy; 6 Interdepartmental Center for Energy and Environment (CIDEA), University of Parma, Parco Area delle Scienze 141/A, 43124 Parma, Italy

**Keywords:** environmental scanning electron microscopy, scanning transmission electron microscopy, energy dispersive X-ray spectroscopy, inductively coupled plasma mass spectrometry, metal sub-microparticles and nanoparticles, food safety, wheat

## Abstract

Metal sub-microparticles (SMPs) and nanoparticles (NPs) presence in food is attributable to increasing pollution from the environment in raw materials and finished products. In the present study, a multifaceted analytical strategy based on Environmental Scanning Electron Microscopy and High-Angle Annular Dark-Field—Scanning Transmission Electron Microscopy coupled with Energy-Dispersive X-ray Spectroscopy (ESEM-EDX, HAADF-STEM-EDX) and Inductively Coupled Plasma Mass Spectrometry (ICP-MS) was proposed for the detection and characterization of metal and metal-containing SMPs and NPs in durum wheat samples, covering a size measurement range from 1 nm to multiple µm. ESEM-EDX and ICP-MS techniques were applied for the assessment of SMP and NP contamination on the surface of wheat grains collected from seven geographical areas characterized by different natural and anthropic conditions, namely Italy, the USA, Australia, Slovakia, Mexico, Austria, and Russia. ICP-MS showed significant differences among the mean concentration levels of metals, with the USA and Italy having the highest level. ESEM-EDX analysis confirmed ICP-MS concentration measurements and measured the highest presence of particles < 0.8 µm in size in samples from Italy, followed by the USA. Less marked differences were observed when particles < 0.15 µm were considered. HAADF-STEM-EDX was applied to a selected number of samples for a preliminary assessment of internal contamination by metal SMPs and NPs, and to expand the measurable particle size range. The multifaceted approach provided similar results for Fe-containing SMPs and NPs. ICP-MS and ESEM-EDX also highlighted the presence of a significant abundance of Ti- and Al-containing particles, while for STEM-EDX, sample preparation artifacts complicated the interpretation. Finally, HAADF-STEM-EDX results provided relevant information about particles in the low nm range, since, by applying this technique, no particles smaller than 50 nm were observed in accordance with ESEM-EDX.

## 1. Introduction

Durum wheat grains, which are used for the production of many baked goods and pasta, are the main energy and vegetable nutrient sources for human nutrition globally [1,2]. Due to their widespread average consumption, durum wheat grains are under close surveillance, becoming a food safety concern as they represent a significant source of food-borne contaminants, the main ones being mycotoxins, toxic trace elements, and pesticides [2]. When grains are milled into flour, processing can also introduce further contamination: contaminants in flour can derive from natural sources, as they can occur naturally in the environment, or originate from anthropogenic sources and even be introduced during various stages of food production, processing, or transport [3,4,5]. The discovery of new sources of contamination or new treatment technologies identifies a contaminant as emerging. Among the emerging contaminants (newly formed or prominent) that threaten environmental health, there is a long list of contaminants of concern, including nanoparticles (NPs) [6].

A regulatory framework is put in place in the European Union to ensure a high level of the consumer’s safety and environmental protection when it comes to food. The current principles of EU legislation on food contamination are laid down in the Commission Regulation (EU) 915/2023 [7,8]. In 2021, the European Food Safety Authority (EFSA) updated the guidance on risk assessment arising from the application of nanoscience and nanotechnologies in the food and feed chain, and human and animal health [9]. This guidance applies also to particles requiring a nanospecific approach to risk assessment in conventional materials that do not meet the definition of engineered nanomaterial set out in the Novel Food Regulation (EU) 2015/2283. This covers the application areas within EFSA’s remit, including novel foods, food contact materials, food/feed additives, and pesticides. Contaminants in (nano)particle form are not specifically addressed in the legislation on food contamination or in the EFSA’s guidance. In the United States, the Food and Drug Administration (FDA) dedicated a task force within the Nanotechnology Program to study the relationship between nanomaterials and food safety. The regulatory framework surrounding the topic of contaminating metal NPs in food products is still evolving and there is a need for comprehensive risk assessment guidelines and regulatory standards to ensure the safety of NPs occasionally present as contaminants [9].

Therefore, in this context, NPs can be considered emerging contaminants, for which numerous issues have not yet been fully addressed within the scientific community, such as the assessment of their toxicity and exposure risk, as well as the development of reliable analytical methods able to characterize and quantify NPs in food and environmental samples [5,6,10,11,12]. In particular, metal and metal-containing NPs can be produced by natural phenomena, such as volcanic ash emissions or biological processes, or derive from anthropogenic activities, having toxic effects both on terrestrial and aquatic ecosystems [13,14]. The presence of NPs in the environment, due to their persistence in soil, water, and air, determines an influence on ecosystems and a potential situation of increasing pollution in raw materials and, subsequently, finished food products. The release of NPs into the environment can therefore pose a high risk of human exposure to NPs through the food chain. 

In particular, the presence of metal NPs contaminating food products may undermine consumer acceptance and lead to a loss of market confidence in the near future, while still posing a potential food safety risk in itself. In the coming years, both these reasons will push the food industry more and more to develop monitoring and prevention strategies for this type of risk. From this perspective, food products that are highly dependent on raw materials exposed to direct atmospheric fall-out contamination are particularly sensitive to the problem.

As stated above, to support regulatory actions, analytical methods for the accurate measurement and characterization of metal NPs in foods are needed, validation being the key activity to assure the traceability and the reliability of the results [15]. The characterization, identification, and quantification of nanomaterials as target species in real samples is a recent challenge that analytical chemistry faces within Analytical Nanoscience and Nanotechnology evolution [16]. In this context, it has to be noted that the isolation of NPs from complex food and environmental matrices is challenging and represents a bottleneck for the development of reliable analytical procedures, given the high heterogeneity in the composition and the polydispersity in the size and shape of the NPs present as contaminants, the very low concentration levels, and the dynamic processes leading to the alteration in the physicochemical properties of NPs [17]. 

Furthermore, a careful sample preparation is crucial to overcome potential artifacts, interferences, and cross-contaminations [15], and avoid the introduction of undesired modifications of NP shape and structure [18,19], since the physical and chemical properties of NPs are determining factors of their toxicities. To date, only a limited number of methods are available in the literature for the characterization and measurements of metals and metal-containing (nano)particles in foods, including wheat [18,20,21,22]. Powerful techniques for NP analysis are Scanning Electron Microscopy (SEM) or Environmental SEM (ESEM) coupled with a field-emission electron gun because of their capacity to visualize the NPs and thus obtain information on their size distribution and shape; furthermore, electron microscopy equipped with Energy-Dispersive X-ray (EDX) spectroscopy provides a rapid nondestructive determination of the elemental composition of the sample. Also, inductively coupled plasma-mass spectroscopy (ICP-MS)-based techniques have been exploited for metal NP analysis, such as ICP-MS for the determination of the total metal concentration after sample filtration [21] or single-particle-ICP-MS (spICP-MS) for the measurement of the mass of the elements recorded in the individual particles and particle number concentration [20]. 

Our research group devised a multi-technique-based approach for the qualitative and quantitative analyses of metal microparticles and sub-microparticles (SMPs) in wheat and wheat-based products (durum wheat seeds, wheat seeds, semolina, wheat flour, biscuits, and pasta samples) using ICP-MS and SEM-EDX [22]. In the study, mainly particles containing iron or titanium in a size range from 1 to 100 μm were detected. Furthermore, a decrease in the concentrations of Fe- and Ti-containing particles was observed from wheat samples to flour and from durum wheat to semolina samples, whereas the particle number did not increase from wheat to finished products, i.e., pasta and biscuits. These findings suggested the external contamination of grains as the main source of metal-containing particles. In a subsequent research study, we focused on the development of an analytical approach based on the combination of ICP-MS and ESEM-EDX techniques to investigate the impacts of environmental pollution and manufacturing processes on metal-containing NP contamination along the pasta production chain, from wheat ears to the finished product. NPs containing mainly Fe and Ti with dimensions < 0.15 µm were detected. The study was integrated with the monitoring of fine and ultrafine particulates in the air near the production plant, highlighting an increase in the concentration levels of total particles during the winter period, in particular of fine particulate matter (PM_2.5_), with the majority of Fe-containing particles.

Other authors explored the application of ESEM to directly characterize the size distribution of a range of engineered NPs in complex environmental and food matrices, performing the detection of these particles by ESEM in liquids to a level of 1 mg/L and down to 30 nm [23]. However, it is known that a limitation of ESEM is that it can provide incomplete results in analyses of particles in the nano-range, since the spatial resolution drops to several tens of nm and only a thin surface layer of the sample is subjected to analysis. Conversely, High-Angle Annular Dark-Field—Scanning Transmission Electron Microscopy (HAADF-STEM) is applied for particle characterization at the nanoscale with a spatial resolution below 1 nm [24,25,26,27]. HAADF-STEM imaging can be combined with EDX analysis (HAADF-STEM-EDX), and some studies have demonstrated the potential of this method for the detection and characterization of NPs in food samples at a high resolution [28,29,30].

Furthermore, sample preparation poses significant analytical challenges, even in relatively simple matrices, and can have an influence on particle size distribution measurement results, thus requiring validated and harmonized sample preparation protocols prior to particle size characterization [31].

Based on these considerations and with the aim of pursuing an in-depth investigation of the contamination of the durum wheat food chain using electron microscopy and atomic spectroscopy techniques, in this work, an analytical strategy based on ESEM-EDX and ICP-MS combined with HAADF-STEM-EDX was evaluated to detect and characterize metal SMPs and NPs in durum wheat samples, resulting in a multifaceted approach of complementary methods able to cover the entire size range from 1 nm to multiple µm. The method was applied to samples of durum wheat coming from different geographical regions of the world, which are exposed to different natural and anthropic conditions. The ESEM-EDX technique combined with ICP-MS was applied to seven durum wheat samples for screening the presence of metal SMPs and NPs on the surface of wheat grains by determining the size and elemental composition of both the SMPs and NPs via direct microscopic imaging.

Based on the screening results, HAADF-STEM-EDX was applied to a selected number of samples coming from three different continents for a preliminary assessment of internal contamination as a complementary approach. It applied a standardized validated sample preparation protocol previously developed for the TEM analysis of food products containing a food additive [30].

These electron microscopy-EDX-based approaches were combined with an independent technique, such as ICP-MS, for total element composition, allowing us to acquire complementary data on the dimensional characterization of metal NPs resulting from the external and internal contamination of durum wheat grains and on the concentration of metal particles.

The information obtained in this study, although preliminary, can play a fundamental role both in the implementation of mitigation actions and in improving future quality assessments for regulatory purposes, highlighting the significance of the results for the food industry.

## 2. Results and Discussion

Taking into account the growing concern in recent years about the contamination of foods by engineered NPs and more generally by SMPs and NPs present in foods [32], in our previous studies focusing on the wheat grain chain, we have shown that raw materials are likely to provide the largest contribution to metal- and metal-containing SMP and NP contamination, since the production processes related to the wheat grain chain usually take place under controlled conditions according to rigorous cleaning standards and procedures [18,22].

In order to investigate the relationship between levels of contamination by metallic SMPs/NPs and the geographical area of wheat harvesting, in the present study, an integrated strategy was devised based on the use of ICP-MS, ESEM-EDX and HAADF-STEM-EDX techniques aimed at achieving a preliminary understanding of the level of wheat contamination in different global geographic areas. The multi-technique approach was applied to evaluate both the external and the internal contamination by SMPs and NPs of durum wheat grains from three different continents. The sampling areas were selected based on high wheat productivity, being characterized by climatic and soil conditions favorable to the cultivation of durum wheat. These geographical areas are influenced by various anthropic activities and natural processes, capable of influencing the environmental conditions of wheat growth.

A similar methodological approach combining EM-based techniques (SEM-EDX, TEM) and inductively coupled plasma optical emission spectrometry (ICP-OES) was proposed by Song et al. to detect, characterize, and quantify engineered NPs (i.e., zinc oxide (ZnO) and titanium dioxide (TiO_2_) NPs) in food products, including wheat flour in food packaging materials, pesticides, and other commercial products [33]. These studies also allowed the authors to successfully investigate the physical properties of engineered NPs, such as particle size and shape, using TEM and SEM-EDX, showing that ZnO and TiO_2_ NPs spiked in corn starch, yam starch, and wheat flour could be determined from 0.05 to 1 wt %, proving that NP contamination in foods can be detected and quantified by a combination of techniques, including TEM, SEM-EDX, and ICP-OES. The authors finally outlined that such findings could contribute to the development of systematic methodologies for the detection, characterization, and quantification of NPs in food matrices.

### 2.1. ICP-MS and ESEM-EDX Measurements

The polycarbonate filters from the durum wheat samples were subjected to analysis by ICP-MS and ESEM-EDX to detect and quantify SMPs and NPs and to identify their elemental compositions in order to assess the external environmental contamination of the wheat grain samples.

ICP-MS is shown to be a very useful and highly sensitive analytical technique for the determination of trace elements in samples of different natures, due to its very low detection limits and multi-element capability. Table S1 reports the validation parameters calculated for the ICP-MS method for all the elements investigated. Table 1 shows the results of the ICP-MS analysis of wheat grain samples containing different concentrations of metals detected and present at levels higher than LOQ: Mg, Al, Ti, Ca, Mn, Fe, and Cs were detected in all samples, with Al and Fe being the most abundant elements.

**Figure 1:**
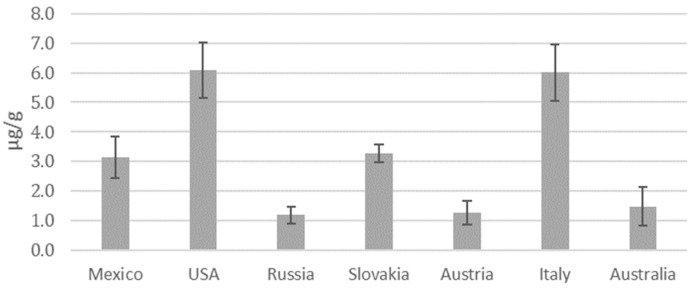
Total metal concentration (µg/g of wheat) measured by ICP-MS analysis, considering the contributions of Mg, Al, Ti, Ca, Mn, Fe, and Cs.

In terms of total metal concentration, some of these elements, such as Al, could be related to the presence of corresponding silicates in the soil of the wheat cultivation field [18]. On the other hand, Cr, Ni, Cu, Zn, and Pb were not detected in any samples. ANOVA showed significant differences (*p* < 0.05) between the mean concentration levels of Mg, Al, Ti, Ca, Mn, Fe, and Cs for the samples from the countries investigated: the USA and Italy are characterized as having the highest level of contamination followed by Mexico and Slovakia, while the lowest levels of contamination were found in samples from Russia, Australia, and Austria (Figure 1). These results are in agreement with the World Air Quality Index ranking of concentration values for up to five key pollutants, including particulate matter (PM_10_) and fine particulate matter (PM_2.5_) (https://waqi.info, last accessed 6 May 2024), according to which Italy and Mexico are recognized as polluted countries on the basis of average levels of fine particulate matter. In particular, the Po Valley (Italy) is densely populated and heavily industrialized, resulting in a large quantity of particulate matter released into the atmosphere as a consequence of various anthropic activities, including biomass burning, vehicular traffic, domestic heating, industry and energy, and agriculture [34,35]. As for the USA, the air quality of the Great Plains region is influenced primarily by industrial production. Similarly, the presence of numerous industries, including those related to the extraction and production of oil and natural gas, in Oklahoma can contribute significantly to air pollution [36]. By contrast, it is known that vast and sparsely populated geographical areas are characterized by reduced pollution levels. As a general comment, it should be considered that, in most cases, air pollution cannot be ascribed to the presence of a single predominant factor: in fact, in the case of countries such as Mexico, a combination of factors, such as population density, transport infrastructures, proximity to desert areas, environmental policies, and agricultural activities, contribute to particulate matter pollution [37,38].

**Figure 2:**
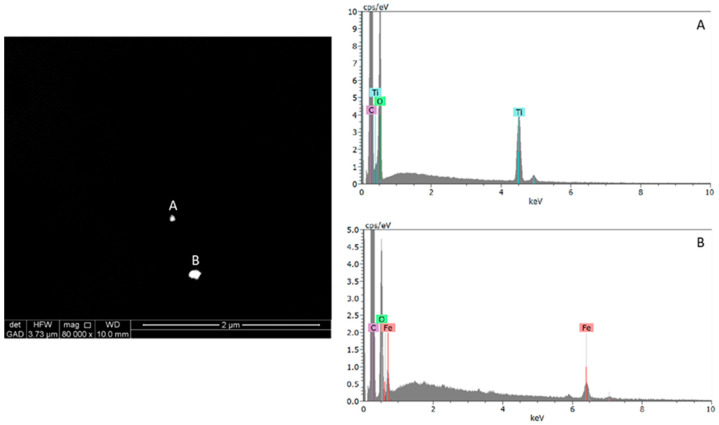
ESEM micrograph (Z-contrast) of (**A**) titanium- and (**B**) iron-containing nanoparticles and the corresponding EDS spectrum of the filter referring to the wheat grain sample from Australia.

Regarding ESEM-EDX particle visualization and counting performed on the polycarbonate membrane sample filters prepared for wheat grain, the metal SMPs and NPs were detected by performing backscatter electron imaging to reveal compositional differences, since the contrast of the backscatter image depends on the average atomic number of the particles [39]. ESEM-EDX analysis of durum wheat sample filters allowed the detection and characterization of individual metal particles in the nanosize range (mainly Ti and Fe) and metal-containing SMPs and NPs (Figure 2). Many different elements contributed to the particulate mass. These results confirm the ICP-MS concentration measurements.

**Figure 3:**
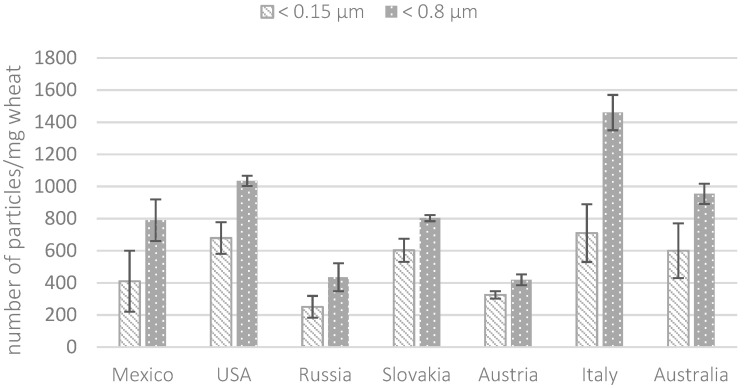
Total number of particles (<0.8 and <0.15 µm) per milligram of wheat counted by ESEM-EDX analysis.

The results obtained by ESEM-EDX in terms of particle counts are shown in Figure 3. Particle counting analysis showed that the highest presence of particles < 0.8 µm in size was observed in wheat samples from Italy followed by the United States, Mexico, Slovakia, and Australia. Less marked differences were observed when particles < 0.15 µm were considered. For Slovakia, Austria, Australia, and the USA the majority (from 63 to 78%) of the counted particles showed sizes smaller than 0.15 µm. The smallest detectable particle size was approximately 50 nm. According to ICP-MS analysis, EDX analysis highlighted a significant abundance of Fe-, Ti-, and Al-containing particles compared to other metals, such as Mn, Cr, and Zn. Fe-containing particles accounted for 42–64% of the total particles, whereas Ti-containing particles ranged from 12 to 24%. Aluminosilicates were observed in all the samples. As observed in a previous study, the presence of metal NPs, such as Fe or Ti, in cereals could result from environmental contamination due to vehicle traffic near the cultivation and production areas [18].

### 2.2. HAADF-STEM-EDX Measurements

As a preliminary assessment, a methodology based on HAADF-STEM imaging and STEM-EDX analysis was developed to detect and identify the potential fraction of metallic SMPs and NPs due to internal contamination inside the grain. The investigation was performed on samples of wheat grains from Italy, Mexico, the USA, and Australia selected as a case study based on the different pollution levels.

A dedicated sample preparation protocol was developed based on the previously standardized and validated protocol for the extraction and subsequent TEM analysis of TiO_2_ particles of the E171 food additive included in food products [30]. The initial centrifugation step allowed cleaning up the sample by removing most of the organic matrix of the plant material. The protocol further dispersed and concentrated the particles such that EM grids containing an amount of well-distributed particles sufficiently high for STEM-EDX analysis could be prepared.

**Figure 4:**
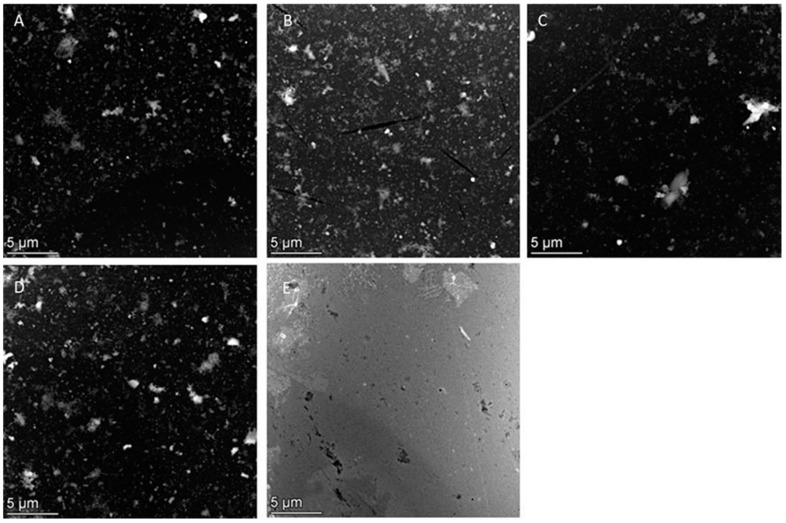
Representative HAADF-STEM images of wheat grain samples from (**A**) Mexico, (**B**) Australia, (**C**) Italy, (**D**) the USA, and (**E**) negative control.

STEM analysis demonstrated the presence of metal-containing particles on the EM grids for all durum wheat samples (Figure 4). The number of particles was substantially larger in the wheat grain samples (Figure 4A–D) than in the negative control sample (Figure 4E). HAADF-STEM-EDX allowed the identification of the subpopulations of (nano)particles by their elemental composition and determining their size, shape, and agglomeration state. In all investigated samples, particles ranging from 50 nm to 1 µm were observed. Although the methodology allows us to concentrate and detect smaller particles [30], it is highlighted that no particles were observed in the lower nano-range (below 50 nm). Additionally, particles with high to low sphericity and more irregular particles were observed.

The elemental composition of the particles observed in the samples included: C-based matrix residue; Fe-containing particles; particles containing P, Ca, Mg, Fe, and Mn; particles containing Ti mostly combined with Al, and in few cases also Fe and Cr; salts (Na, Ca, and K combined with Cl and S); and/or (alumino)silicates (Al, Si, and K) (Table 2).

The Cu and to a lesser extent Si signals observed in all spectra can be attributed to signals originating from the TEM grid (Cu signal) and the EDX detector (Si signal).

**Figure 5:**
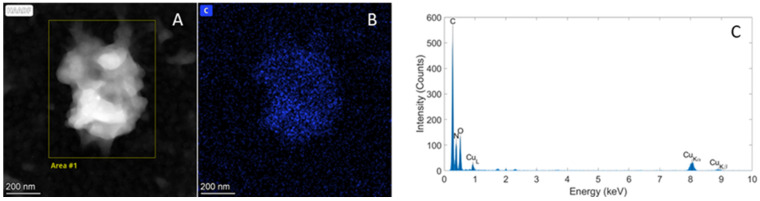
(**A**) HAADF-STEM image showing a particle in the wheat grain sample from the USA. (**B**,**C**) Elemental EDX analysis demonstrating that this particle contains mostly carbon with (**B**) the corresponding spectral image of C and (**C**) the spectrum of the area indicated in (**A**).

Carbon was present in relatively high quantities on specific particles for all wheat samples. As an example, the HAADF-STEM image in Figure 5 shows a particle in the wheat grain sample from the USA.

These particles can most likely be attributed to the remaining organic matrix of the wheat grains not completely removed during the applied purification steps. However, the presence of matrix residues in relative high amounts, identified based on their carbon signal, did not hamper the detection and identification of other particles by STEM-EDX.

**Figure 6:**
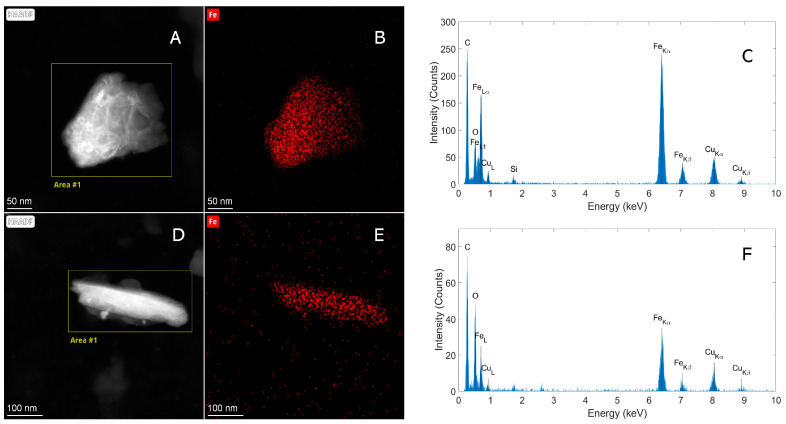
(**A**,**D**) HAADF-STEM images showing particles in wheat grain samples from Mexico and Italy. (**B**,**C**,**E**,**F**) Elemental EDX analysis demonstrating that the particles contain Fe, with (**B**,**E**) the corresponding spectral image of Fe and (**C**,**F**) the spectra of the areas indicated (**A**,**D**).

Fe-containing particles were observed in samples from Italy and Mexico, while in the sample from the USA, a particle containing Sn and Fe was found (Table 2). STEM-EDX analysis showed that multiple physical forms of Fe-containing particles are present in the wheat grain samples (Figure 6).

The STEM-EDX analysis of the particle shown in Figure 6A–C shows, for example, a different iron/oxygen ratio and a different morphology compared to the particle shown in Figure 6D–F. The rod-like morphology (Figure 6D) and the higher oxygen/iron ratio (Figure 6F) are typical for goethite iron hydroxide [40]. No Fe-containing particles were observed in the sample from Australia or in the negative control sample.

**Figure 7:**
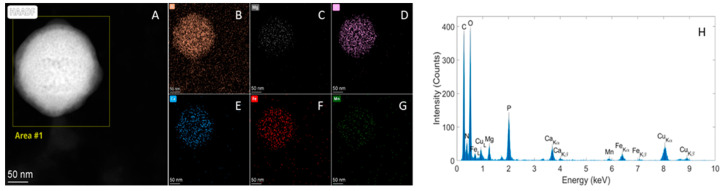
(**A**) HAADF-STEM image showing a particle in the wheat grain sample from Mexico. (**B**–**H**) Elemental EDX analysis demonstrating that this particle contains P, Ca, Mg, Fe, and Mn, with (**B**–**G**) the corresponding spectral images and (**H**) the spectrum of the area indicated in (**A**).

In addition, multi-element spherical particles containing P, Ca, Mg, Fe, and Mn (all elements or some of these elements) were abundantly present in all samples [41], but not in the negative control (Figure 7).

The high level of concentration of particles using centrifugation (250 times) and the high resolution of HAADF-STEM-EDX resulted in the detection of specific populations of particles in the control sample: Ti-Al-containing particles were, for example, observed in all samples, including the control sample (Figure S2, Supplementary Material). These can be classified as artifacts originating from the sonicator probe. Previously, Betts et al. [42] observed that Ti-Al particles, as shown in Figure S2, detach from the tip of the sonicator probe. It was not possible to conclude if any of the observed Ti-Al particles originated from the samples or if they were solely created due to the applied probe sonication method. In few cases, Fe and Cr signals were also measured by EDX on these particles (Table 2). Alternatively, a sonication device, such as a sonication bath, a vial tweeter, or a cup-horn sonicator without direct contact with the sample, could be applied to avoid such contamination in future investigations. In addition, the low amounts of (alumino)silicates observed as a background in the four wheat samples and in the negative control sample possibly originated from the Milli-Q water used for sample preparation [43].

The multifaceted approach provided similar results for Fe-containing SMPs and NPs. It can be hypothesized that the exchanges of the leaves and their stomata with the external atmosphere can involve the transport of NPs toward the ear, with a progressive conglobation of NPs also inside (and not only outside) the grain, also taking into account that many soil and environmental factors combine to regulate the Fe supply to plants, as recently reviewed by Lv et al. [44]. In addition, the Fe-containing particles inside the grains could originate from the soil through root exposure.

## 3. Materials and Methods

### 3.1. Chemicals and Materials 

Ultrapure deionized water (Milli-Q water) was produced using Element A10 equipment (Millipore, Bedford, MA, USA) for ICP-MS and ESEM-EDX analyses, and Arium^®^ pro DI/UV equipment (Sartorius, Göttingen, Germany) for STEM-EDX analysis. The ICP-MS multi-element calibration standard (ICM-630) was purchased from Agilent Technologies Italia (Milan, Italy). Intermediate standard solutions were prepared using 10% (*w*/*v*) nitric acid, while the working standard solutions were diluted daily. Polycarbonate filters, 47 mm in diameter, 0.1 µm in pore size, were from GVS, Bologna, Italy. Minisart NML Syringe filter surfactant-free cellulose acetate (SFCA) (0.2 µm) was from Sartorius (Göttingen, Germany). Liquid scintillation vials were from Wheaton (Millville, NJ, USA).

Samples from different countries were collected through direct collaboration with local colleagues/business partners of Barilla’s factories and commercial offices in different parts of the world, thus ensuring sourcing and origin from selected territories. In particular, durum wheat seeds were collected from seven geographical areas of the world: Yaqui Valley (Sonora, Mexico), Darling Downs district (Queensland, Australia), Po Valley (Italy, Europe), Nitra region (Slovakia, Europe), Burgenland (Austria, Europe), Krasnodar Krai (Russia), and Great Plains (Oklahoma, USA). For each country, a wheat sample representing the annual harvest was obtained: samples from different fields in each selected geographical area were collected and mixed to obtain an aggregate sample in a storage center. Figure S1 illustrates the map of wheat grain sampling sites.

### 3.2. Sample Preparation

A previously developed sample preparation strategy for ICP-MS and ESEM-EDX measurements was applied [18]. Briefly, 2 g of wheat seeds were placed into 100 mL glass tubes and immersed in 40 mL of Milli-Q water under stirring for 1 h. Afterwards, the aqueous fraction was filtered using a 0.1 µm polycarbonate filter, air-dried in Petri dishes, and divided into two parts: one for ICP-MS and the other for ESEM-EDX analysis. Three independent replicates of the whole procedure were performed for each sample. Technical blanks were prepared in triplicate by processing Milli-Q water. The laboratory was set up to avoid external contamination by using only metal-free items and working under a laminar flow hood.

In the case of STEM-EDX analysis, 12 g of seeds were weighed in a 50 mL polypropylene disposable tube and 12 mL of Milli-Q water were added. The sample was sonicated using an XUBA3 ultrasonic bath (Grant Instrument, Cambridge, UK) at maximum power for 1 h. The material was dried and crushed using a mortar. An amount of 0.7 g of crushed raw material was weighed and placed in 15 mL of Milli-Q water. To reduce centrifugation times, the suspension was divided in two parts: two 50 mL polypropylene disposable tubes were filled each with a volume of 5–6 mL transferred from the suspension and centrifuged at 7759× *g* (speed: 7830 rpm) for 4 h using a 5430R centrifuge (Eppendorf, Hamburg, Germany) equipped with Rotor FA-35-6-30. Centrifugation times were calculated based on Stoke’s law using the density of anatase TiO_2_, such that the particles with a density higher than 3.89 g/cm^3^ and larger than 30 nm were spun down quantitatively [30]. After centrifugation, the supernatants were removed and both pellets were re-suspended in 5.5 mL of Milli-Q water, filtered with SFCA 0.2 µm syringe filters, and subsequently re-combined in a 20 mL liquid scintillation vial. To reduce agglomeration, the sample was dispersed by probe sonication delivering 7 kJ of energy using a calibrated Vibracell™ 75,041 ultrasonifier (Fisher Bioblock Scientific, Aalst, Belgium) equipped with a 13 mm probe at 20% amplitude. The sample was cooled in ice water during sonication. The device was calibrated according to NANoREG D4.12 SOP Probe Sonicator Calibration for ecotoxicological testing [45]. A second centrifugation step to concentrate the sample was performed under the same experimental conditions as the first centrifugation step, except that, after centrifugation, both pellets were re-suspended in 500 μL of Milli-Q water and re-combined in a 1.5 mL microcentrifuge tube. A third centrifugation step was applied using Rotor FA-45-24-11-HS to further concentrate the sample. Microcentrifuge tubes were centrifuged for 1 h at 30,184× *g* (speed 17,500 rpm). The supernatant was removed, and the pellets were each re-suspended in 20 μL of Milli-Q water. TEM specimens (grids) were prepared by using the grid-on-drop deposition method on Alcian blue pre-treated carbon and pioloform-coated 400-mesh copper TEM grids (Agar Scientific, Stansted, Essex, UK) based on the SOP “Preparation of EM-grids containing a representative sample of a dispersed nanomaterial” [31]. In addition to the durum wheat samples, a sample containing only 15 mL of Milli-Q water was prepared as a negative control following the same procedure, including all centrifugation and probe sonication steps.

### 3.3. ICP-MS Analysis

ICP-MS measurements were carried out by X7 series II ICP-MS (ThermoFisher, Bremen, Germany) equipped with a CETAC AS-500 autosampler (CETAC, Omaha, NE, USA) operating in flow injection full-scan acquisition mode and daily calibrated with the autotune procedure. The quantitation of metals by ICP-MS was performed according to the protocol previously described [22]. Method validation for the ICP-MS analysis of the detected elements, namely Cs, Mg, Ca, Al, Ti, Cr, Mn, Fe, Ni, Cu, Zn, and Pb, was performed according to EURACHEM guidelines [46]. Detection (y_D_) and quantitation (y_Q_) limits were expressed as signals based on the mean blank (${\bar{x}}_{b}$) and the standard deviation of blank responses ($s_{b}$) as follows:

(1) $$y_{D}={\bar{x}}_{b}+2ts_{b}$$

(2) $$y_{Q}={\bar{x}}_{b}+10t$$

where *t* is the constant of the Student’s t-distribution (one-tailed), depending on the confidence level and degrees of freedom. A 95% confidence level was chosen. ${\bar{x}}_{b}$ and $s_{b}$ were calculated performing ten blank measurements, using a polycarbonate filter that did not come into contact with the samples as the blank matrix. The concentration values of the detection limit (LOD) and quantification limit (LOQ) were obtained by the projection of the corresponding signals,$\;y_{D}$ and $y_{Q}$, through a calibration plot, y = f(x), onto the concentration axis. LOD and LOQ values are expressed as ng per g of wheat grains. Linearity was established over two orders of magnitude by analyzing eight concentration levels covering a range from the LOQ of each element, performing three replicate measurements for each level and applying Mandel’s fitting test. The significance of the intercept (significance level 5%) was established by running a Student’s *t*-test.

Intra-day repeatability and intermediate precision were calculated in terms of RSD% for each element, performing six replicate measurements at low, middle, and high concentration levels over the corresponding linearity range. Intermediate precision was estimated over 3 days verifying the homoscedasticity of the data and performing the analysis of variance (ANOVA) at a confidence level of 95%.

Trueness was evaluated in terms of recovery rate by spiking procedure at low, middle, and high concentration levels over the linearity range, and calculated as the ratio of the concentration of analyte found to that added.

### 3.4. ESEM-EDX Analysis

A Quanta™ 250 by FEG (FEI, Hillsboro, OR) equipped with QUANTAX XFlash^®^ 6|30 detector for energy-dispersive X-ray spectrometry (Bruker Nano GmbH, Berlin, Germany) was used with the sample chamber at 70 Pa. Sample filters were analyzed by recording the backscattered electron (BSE) signal (Z-contrast) using the gaseous backscattered analytical (GAD) detector. EDX spectrum was acquired for every bright particle with a diameter smaller than 0.8 µm. Particle counting was performed by exploring three different zones of 0.12 mm^2^ by acquiring 225 adjacent BSE images (1024 × 884 pixels; horizontal field of view, 24.9 µm) each, for a total evaluated area of 0.36 mm^2^. Images were recorded using ESPRIT’s Jobs function, while the localization of the bright particles, the acquisition of EDX spectra, and the chemical classification were performed manually.

### 3.5. HAADF-STEM-EDX Analysis

HAADF-STEM imaging and EDX analyses were performed using a 200 kV Talos F200S G2 TEM equipped with an HAADF detector and a Super-X EDS detector consisting of 2 windowless silicon drift detectors (SDDs), using Velox software (Version 2.10) (Thermo Fisher Scientific, Eindhoven, The Netherlands). STEM images were recorded with a scan size of 1024 × 1024 pixels, a dwell time of 20 µs, a probe convergence angle of 7.5 mrad, and a camera length of 260 mm. Based on the analysis of a series of representative images recorded at high, intermediate, and low magnifications, and covering the entire specimen, the properties of the nano-objects of interest, such as size, morphology, and agglomeration state, were demonstrated.

EDX spectral imaging was performed using a dwell time of 20 µs and a live time of 2.1 or 4.2 min, where 10 or 20 frames were recorded, respectively. EDX conditions were optimized so that background signals could be interpreted and that beam-induced transformations of the particles were minimal.

### 3.6. Statistical Analysis

All statistical analyses, including one-way ANOVA analysis at the confidence level of 95%, were carried out using MATLAB R2023a.

## 4. Conclusions

A multi-method approach combining complementary techniques, such as ICP-MS, ESEM-EDX, and HAADF-STEM-EDX, was successfully proposed for the detection of metal and metal-containing SMPs and NPs in durum wheat grain samples.

ICP-MS showed significant differences between the mean concentration levels of metals for the samples from the countries investigated, with the USA and Italy having the highest level of contamination. ESEM-EDX analysis confirmed ICP-MS concentration measurements, especially for particles < 0.8 µm in size, while less marked differences were observed for particles < 0.15 µm. The smallest detectable particle size was approximately 50 nm. The developed method, based on the high-resolution HAADF-STEM-EDX technique (1 nm) associated with an adequate sample treatment for particle pre-concentration and a representative deposition on the EM grid, permitted us to extend the size range. No particles were observed in the lower nano-range, i.e., below 50 nm by ESEM and STEM.

The multifaceted approach provided similar results for Fe-containing SMPs and NPs, assuming the foliar and root uptake of particulate air contaminants or soil constituents. ICP-MS and ESEM-EDX also highlighted a significant abundance of Ti- and Al-containing particles, while for STEM-EDX, sample preparation artifacts (the detachment of Ti-Al particles from the sonicator probe) complicated the interpretation of Ti- and Al-containing particles. It is doubtful whether Ti-containing contaminants are also present inside the grains.

The application of the proposed analytical strategy that produces concordant results emphasizes the utility of a multifaceted analytical approach for the assessment of metal SMP and NP contamination of wheat grains or other raw materials.

Finally, the information achieved in this study, regardless of the specific geographical area, can play a pivotal role both in implementing mitigation actions and in improving future quality assessments for regulatory purposes. Considering nanomaterials as target species has led to the definition of analytical nanometrology; in this context, the lack of standardized or reference materials as well as the harmonization of validation procedures are the main issues to be addressed in the near future to assure the traceability, comparability, and quality of the results.

## Figures and Tables

**Table 1:** Element concentrations (µg/g wheat) determined by ICP-MS analysis (n = 3).

	Mexico	USA	Russia	Slovakia	Austria	Italy	Australia
Mg	0.36 ± 0.05	0.9 ± 0.1	0.20 ± 0.05	0.44 ± 0.08	0.31 ± 0.07	0.8 ± 0.1	0.10 ± 0.05
Al	1.2 ± 0.2	2.2 ± 0.4	0.4 ± 0.1	1.2 ± 0.1	0.40 ± 0.04	2.4 ± 0.3	0.7 ± 0.2
Ti	0.06 ± 0.02	0.12 ± 0.02	0.03 ± 0.01	0.06 ± 0.01	0.05 ± 0.02	0.10 ± 0.02	0.03 ± 0.01
Ca	0.3 ± 0.1	0.7 ± 0.1	0.21 ± 0.06	0.3 ± 0.1	0.2 ± 0.1	0.4 ± 0.1	0.2 ± 0.1
Mn	0.04 ± 0.01	0.06 ± 0.01	0.025 ± 0.001	0.04 ± 0.01	0.014 ± 0.003	0.05 ± 0.01	0.03 ± 0.01
Fe	1.0 ± 0.4	1.8 ± 0.2	0.4 ± 0.1	1.06 ± 0.03	0.3 ± 0.1	2.0 ± 0.4	0.4 ± 0.3
Cs	0.16 ± 0.02	0.34 ± 0.04	0.032 ± 0.008	0.13 ± 0.01	0.04 ± 0.01	0.26 ± 0.04	0.1 ± 0.03
Tot	3.1 ± 0.7	6.1 ± 0.9	1.2 ± 0.3	3.3 ± 0.3	1.3 ± 0.4	6.0 ± 1.0	1.5 ± 0.6

**Table 2:** Description of the durum wheat raw materials and results obtained by HAADF-STEM-EDX analysis.

	Mexico	USA	Italy	Australia	Negative Control
C-based matrix residue	C-based matrix residue present	C-based matrix residue present	C-based matrix residue present	C-based matrix residue present	-
Fe-containing particles	Fe-containing particles present	One particle containing Sn and Fe present	Fe-containing particles present; multiple types (morphologies) observed	-	-
Particles consisting of a combination of P, Mg, Ca, Mn, and Fe	Particles containing Mg, P, Ca, Mn, and Fe present	Particles containing Mg, P, Ca, Mn, and Fe present	Particles containing P, Ca, and Fe present	Particles containing Mg, P, Ca, Mn, and Fe present	-
Silicates or aluminosilicates	-	Silicates and aluminosilicates present	Silicates present	Silicates present	Aluminosilicates present
Ti-Al-based particles	Ti-Al-based particles present	Ti-Al-based particles present; few also containing Fe and Cr	Ti-Al-based particles present; few also containing Fe and Cr 1 particle containing Si, Ti, and Al	Ti-Al-based particles present; few also containing Fe and Cr	Ti-Al-based particles present

## Data Availability

Data are contained within the article or Supplementary Materials.

## Supplementary Materials

The following supporting information can be downloaded at: https://www.mdpi.com/article/10.3390/molecules29133148/s1, Figure S1: Map showing the seven wheat grain sampling sites investigated in this study. Figure S2: (A) Low-magnification HAADF-STEM image of region containing many Ti-Al particles, most probably originating from the sonicator probe; (B) HAADF-STEM image showing a particle in the wheat grain sample from the USA, most probably originating from the sonicator probe. (C–E) Elemental EDX analysis demonstrating that this particle consists of Ti and Al, with (C, D) the corresponding spectral images and (E) the spectrum of the area indicated in B; Table S1: Validation parameters calculated for the ICP-MS method.
