# Supplementary material for: The Combined ICP-MS, ESEM-EDX, and HAADF-STEM-EDX Approach for the Assessment of Metal Sub-Micro- and Nanoparticles in Wheat Grain
Source: Molecules. 2024 Jul 2;29(13):3148. doi: 10.3390/molecules29133148 (PMC11243335; doi:10.3390/molecules29133148)
Supplement: Supplementary file 1 [file molecules-29-03148-s001.zip › molecules-3061441-supplementary.pdf]

Supplementary material

The Combined ICP-MS, ESEM-EDX, and HAADF-STEM-EDX Approach for the Assessment of Metal Sub-Micro- and Nanoparticles in Wheat Grain

Maurizio Piergiovanni [1], Monica Mattarozzi [1,2,*], Eveline Verleysen [3], Lisa Siciliani [3], Michele Suman [4,5], Federica Bianchi [1,6], Jan Mast [3] and Maria Careri [1,2,*]

[1] Department of Chemistry, Life Sciences and Environmental Sustainability, University of Parma, Parco Area delle Scienze 17/A, 43124 Parma, Italy; maurizio.piergiovanni@unipr.it (M.P.); federica.bianchi@unipr.it (F.B.)
[2] Interdepartmental Center on Safety, Technologies and Agri-Food Innovation (SITEIA.PARMA), University of Parma, Parco Area delle Scienze 181/A, 43124 Parma, Italy
[3] Service Trace Elements and Nanomaterials, Sciensano, Groeselenbergstraat 99, 1180 Uccle, Belgium; eveline.verleysen@sciensano.be (E.V.); lisa.siciliani@sciensano.be (L.S.); jan.mast@sciensano.be (J.M.)
[4] Advanced Laboratory Research, Barilla G. e R. Fratelli S.p.A., Via Mantova, 166, 43122 Parma, Italy; michele.suman@barilla.com
[5] Department for Sustainable Food Process, Catholic University Sacred Heart, via Emilia Parmense 84, 29122 Piacenza, Italy
[6] Interdepartmental Center for Energy and Environment (CIDEA), University of Parma, Parco Area delle Scienze 141/A, 43124 Parma, Italy
* Correspondence: monica.mattarozzi@unipr.it (M.M.); maria.careri@unipr.it (M.C.)

Table S1. Validation parameters calculated for the ICP-MS method.

	LOD (ng/g)	LOQ (ng/g)	Intra-day repeatability (RDS %)[a]	Intermediate precision (RSD%)[b,c]	Linearity range (µg/g)	Recovery rate (%)
Mg	24	78	9%	14%	LOQ-10	95±1 - 103±2
Al	72	240	11%	16%	LOQ-25	94±1 - 104±2
Ti	6	22	6%	11%	LOQ-5	96±1 - 103±1
Ca	86	200	11%	15%	LOQ-25	92±1 - 105±2
Mn	1	3.2	9%	13%	LOQ-0.5	94±2 - 102±2
Fe	45	150	12%	18%	LOQ-25	97±1 - 102±2
Cs	10	32	7%	10%	LOQ-5	92±1 - 103±2
Cr	20	58	9%	13%	LOQ-10	94±2 - 104±1
Ni	3.2	10.6	10%	16%	LOQ-5	98±1 - 103±1
Cu	3.4	11	6%	13%	LOQ-5	93±2 - 105±1
Zn	54	180	9%	15%	LOQ-25	93±1 - 103±2
Pb	24	78	7%	13%	LOQ-10	92±2 - 101±1

[a] $n=6$
[b] $n=18$
[c] $p > 0.05$, ANOVA analysis

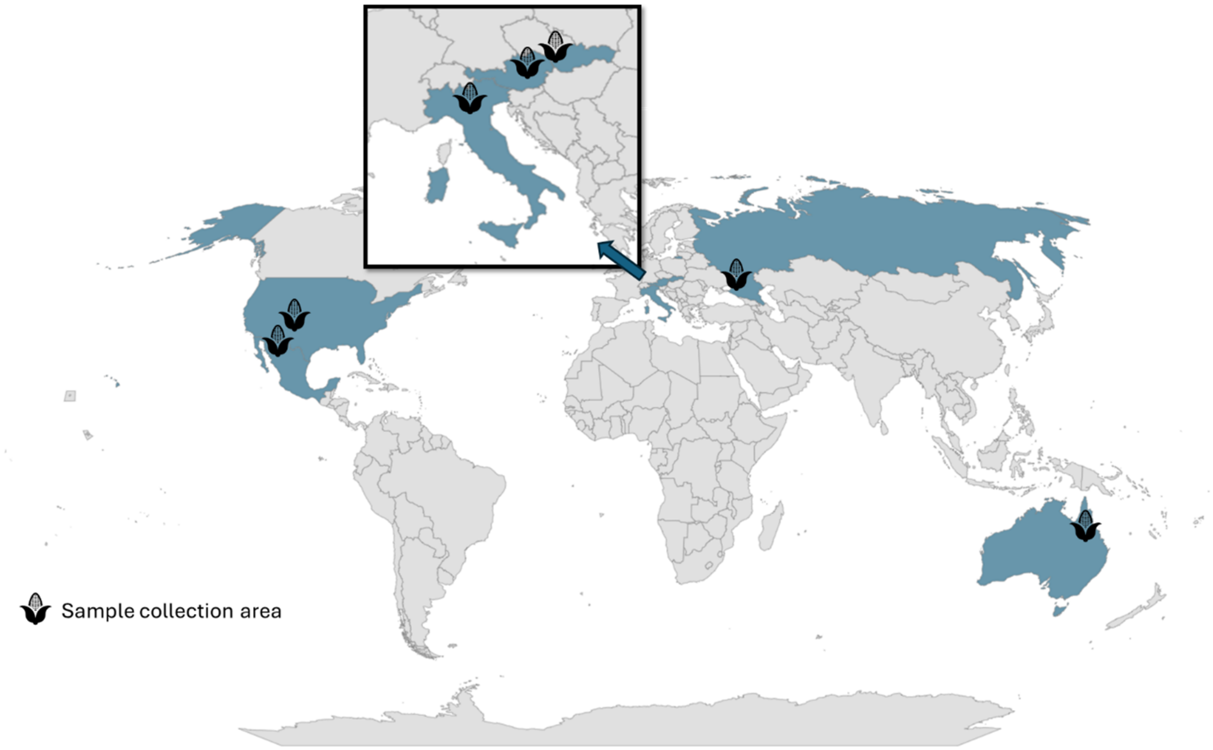

Figure S1. Map showing the seven wheat grain sampling sites investigated in this study.

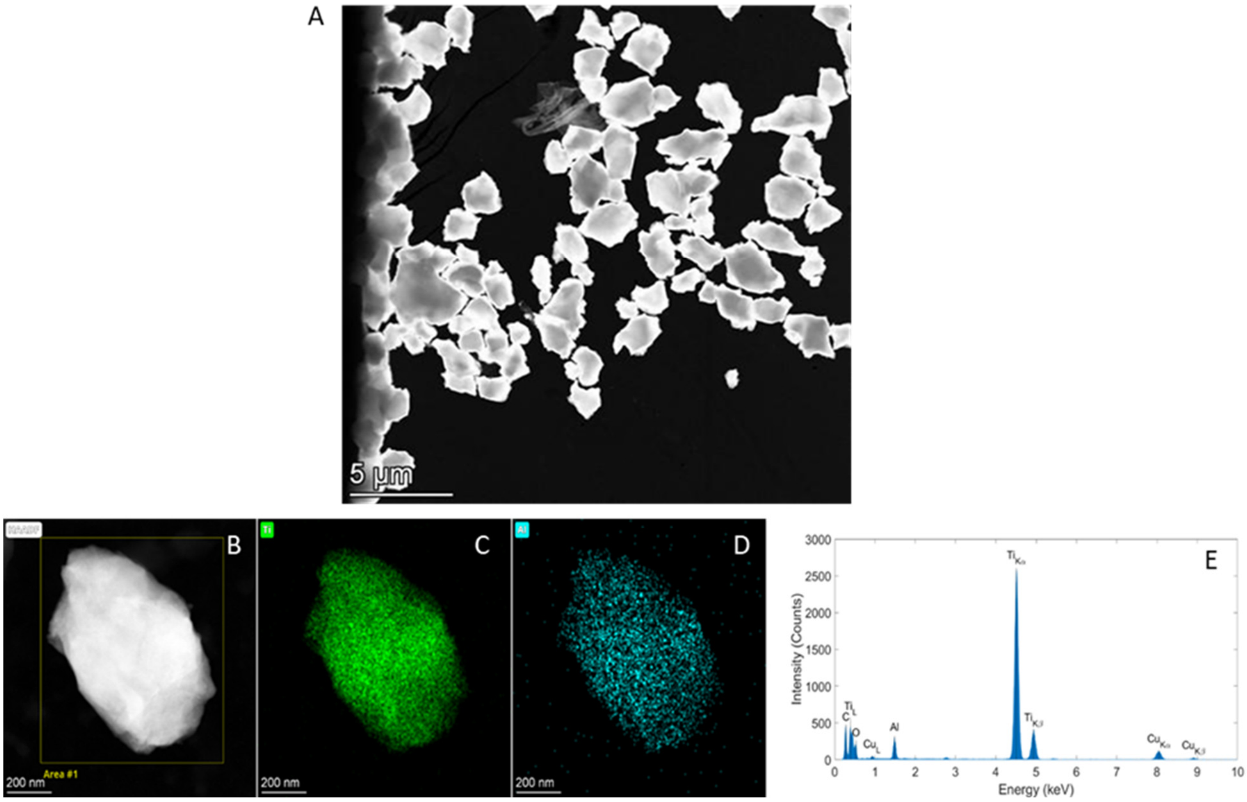

Figure S2. (**A**) Low magnification HAADF-STEM image of region containing many Ti-Al particles, most probably originating from the sonicator probe; (**B**) HAADF-STEM image showing a particle in wheat grain sample from the USA, most probably originating from the sonicator probe. (**C-E**) Elemental EDX analysis demonstrating that this particle consists of Ti and Al, with (**C, D**) the corresponding spectral images and (**E**) the spectrum of the area indicated in (**B**).
